# Rationale and methods for the Exercise for Type 1 Diabetes Education program: a pilot randomized controlled trial of an education program to support adults with type 1 diabetes mellitus (T1DM) to undertake exercise

**DOI:** 10.1136/bmjdrc-2019-000693

**Published:** 2019-12-30

**Authors:** Parth Narendran, Niamh Quann, Dinesh Nagi, Ian Gallen, Janet Gorton, Heather Daly, Catherine Thompson, Nishal Bhupendra Jaicim, Melanie Davies, Robert C Andrews

**Affiliations:** 1 Institute of Immunology and Immunotherapy, University of Birmingham, Birmingham, UK; 2 Department of Diabetes, University Hospitals Birmingham NHS Foundation Trust, Birmingham, UK; 3 Leicester Clinical Trials Unit, University of Leicester, Leicester, UK; 4 Edna Coates Diabetes and Endocrine Unit, Mid Yorkshire Hospitals NHS Trust, Wakefield, UK; 5 Diabetes and Endocrinology, Royal Berkshire NHS Foundation Trust, Reading, UK; 6 Department of Endocrinology and Diabetes, Taunton and Somerset NHS Foundation Trust, Taunton, UK; 7 Diabetes Research Centre, University of Leicester, Leicester, UK; 8 Department of Diabetes, Taunton and Somerset NHS Foundation Trust, Taunton, UK; 9 Institute of Biomedical and Clinical Sciences, University of Exeter, Exeter, Devon, UK

**Keywords:** exercise, education, hypoglycemia, type 1

## Abstract

**Objective:**

Regular exercise in people with type 1 diabetes mellitus (T1DM) can result in considerable improvements in health and reduction in cardiovascular events and death. However, a large proportion of people with T1DM are not active. Fear of hypoglycemia and lack of knowledge on how to manage their diabetes are major barriers to exercise in people with T1DM, but few patients receive specific advice about how to adjust insulin and carbohydrate for activity. Furthermore, healthcare professionals (HCP) currently lack the knowledge to advise patients on how to manage their diabetes when active and would like formal training in exercise prescription for people with T1DM.

**Research design and methods:**

This study is divided into two stages. The first stage develops an education program aimed to support people with T1DM to exercise using the Medical Research Council framework. The second stage is a pilot randomized controlled trial (RCT) that aims to collect the key variables to design a definitive trial to test the efficacy and cost-effectiveness of the education package. We aim to recruit 96 patients with T1DM at two UK hospitals.

**Conclusions:**

This article outlines the protocol for a pilot RCT to develop a program of education that will support adults with T1DM to undertake safe and effective exercise. This is accompanied by training for HCPs to deliver this educational intervention. Successful completion of this program of work will address some of the barriers to exercise in adults with T1DM, and should facilitate an increase in exercise for this group of people.

**Trial registration number:**

ISRCTN61403534.

**Significance of this study**

**What is already known about this subject?**

- Adults with type 1 diabetes mellitus (DM) undertake low levels of exercise. This is in part due to lack of confidence in managing their diabetes when exercising.
- Currently there is no dedicated structured education program that supports the management of type 1 DM for exercise.

**What are the new findings?**

- The objective of the Exercise for Type 1 Diabetes Education study is to develop and pilot an education program in the UK for people with type 1 DM (with accompanying training for healthcare professionals (HCP) to deliver this program) to guide insulin and carbohydrate adjustment for safe exercise.

**How might these results change the focus of research or clinical practice?**

- The program can be delivered by trained HCPs, meaning it can be embedded into the routine care setting.
- If demonstrated to be effective, this program could potentially expand currently available support for adults with type 1 DM who wish to exercise.

## Introduction

### Background

Patients with type 1 diabetes mellitus (T1DM) who exercise regularly have to take less insulin, have a better lipid profile and are more likely to reach their HbA1c and blood pressure targets than inactive patients.^1 2^ They are also more likely to have a better quality of life.^1^

Based on these findings, guidelines recommend that all adults with T1DM accumulate 150 min/week of moderate to vigorous aerobic exercise with no more than 2 consecutive days of doing no activity.^3^

### How active are patients with T1DM?

Many people with T1DM struggle to meet the recommended levels of exercise. Sixty-three percent of German and Austrian people with T1DM, in a cross-sectional study, reported doing no regular physical activity (PA).^4^ Similar results were found in a Finnish study, where 43% of people with T1DM were doing less than one session of PA per week.^5^ Studies in which PA has been measured objectively have found similar findings. In a Canadian study only 43% of women and 55% of men with T1DM were active.^6^ In a UK study, newly diagnosed adults with T1DM spent a quarter less time in moderate to vigorous PA per day than healthy matched controls.^7^

### What are the barriers to exercise in people with T1DM?

Normally when exercising, changes in insulin and counter-regulatory hormone secretion are made which are dependent on the type of exercise being performed. These changes facilitate an increase in liver glucose production, which matches skeletal muscle glucose uptake during exercise.^8^ A change in the secretion of these hormones is also seen after activity to facilitate recovery and adaptation to the exercise. As a result of these changes, blood glucose levels remain stable before, during and after exercise.

In T1DM, fuel regulation is difficult as the insulin level does not fall in response to exercise and there may be impaired secretion or action of counter-regulatory hormones, making normal fuel regulation difficult. After activity the inability of the pancreas to increase insulin if needed, and reduced blood concentrations of counter-regulatory hormones can hamper recovery and adaptation to exercise. This means that hypoglycemia both during and following exercise becomes a significant risk. Furthermore, hyperglycemia prior to, and following, some types of exercises can also be problematic.^9^ In order to prevent these problems patients with T1DM need to make changes to their insulin dosages and nutrition to try and mimic the normal physiological responses seen with the exercise they are undertaking. This requires a lot of skill and extensive knowledge.

In people with T1DM many of the barriers, motivators and facilitators to PA are similar to the general public, such as lack of time, work pressures and bad weather.^10^ In addition, they worry about having low blood glucose during exercise and how they should adjust their insulin and carbohydrate intake to keep glucose stable around exercise. Improved knowledge on how insulin works and education on how to minimize high and low blood glucose excursions with exercise helps reduce these anxieties. Reluctance of physicians to recommend exercise to people with T1DM can also be a barrier.^11^

### Where do patients with T1DM obtain information about diabetes management when exercising?

No national validated education program for people with T1DM around exercise exists, which means that patients must obtain information on this subject from their healthcare workers, internet sites, books, and pamphlets. Information from these sources tends to be generic and not detailed enough to take into account the precautions required for differing type, duration and intensity of exercise. Similarly, no validated courses exist for healthcare professionals (HCP) to learn how to manage and support patients on nutritional adjustments for diabetes and activity. Thus they are left to obtain information from conference lectures, journals, books and websites. Again this advice tends to be generic and insufficiently detailed. Not surprisingly, many HCPs who work regularly with patients with T1DM feel they lack the knowledge and confidence to advise patients on strategies to manage their diabetes when undertaking exercise.

In light of this, we undertook an internet survey to try and understand the knowledge levels of HCPs who were giving advice about activity to people with T1DM. In this survey of 252 HCPs, knowledge levels were poor. For example, two-thirds did not know what advice to give patients on what to do with their short-acting or long-acting insulin when active and two-thirds were unable to identify the time point at which patients were most likely to experience hypoglycemia with different sports. A total of 91% of HCPs felt they would like formal training in exercise prescription for people with T1DM.^12^

### Overarching aim

The overarching aim of this study is to support safe exercise for people with T1DM. The specific aim is to develop and pilot an education program for such people (with accompanying training for HCPs to deliver this program) to guide insulin and carbohydrate adjustment for safe exercise. The results will be used to design a definitive trial to assess the effect of this education program on exercise levels in people with T1DM.

### Study design and specific outcomes

**Figure 1:**
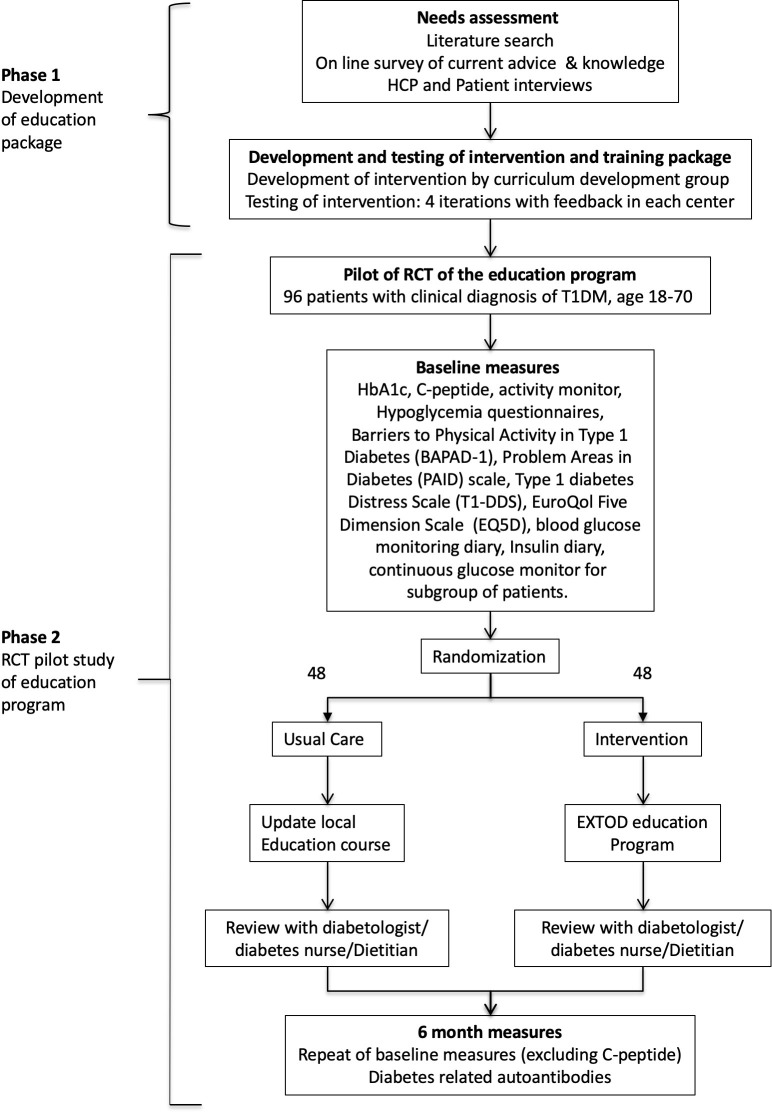
Study flow chart. HCP, healthcare professional; RCT, randomized controlled trial; T1DM, type 1 diabetes mellitus.

This is a multicenter study, divided into two phases (see study flow sheet, figure 1).

In phase 1, the primary aim is to develop an education program for patients with T1DM and accompanying training for HCPs who regularly work with patients with T1DM to guide insulin and carbohydrate adjustment for safe exercise.

In phase 2, the primary aim is to conduct a pilot randomized controlled trial (RCT) to:

1. Determine the number of people with T1DM who would be eligible to participate in an RCT of such an education program.
2. Determine the proportion of these people who would be willing to participate in this trial (ie, recruitment rate), and their characteristics.
3. Define the rates of adherence to the intervention and participant dropout from the study, particularly to determine whether retention differs between the usual care and intervention arms.
4. Generate estimates of statistical properties of potential outcome measures (eg, variances) that are needed for sample size calculations for the definitive trial. The outcome measures that will be assessed are exercise, fear of hypoglycemia, frequency of hypoglycemia, self-reported barriers to exercise, and well-being.

We will also pilot methods for collecting outcome measures and assess the acceptability of these outcome measures. We will also validate our processes for recruitment, randomization, treatment, and follow-up assessments. Information from phase 2 will enable us to design with confidence a definitive RCT to assess the effect of this education program in patients with T1DM.

## Methods

### Methods for phase 1

**Figure 2:**
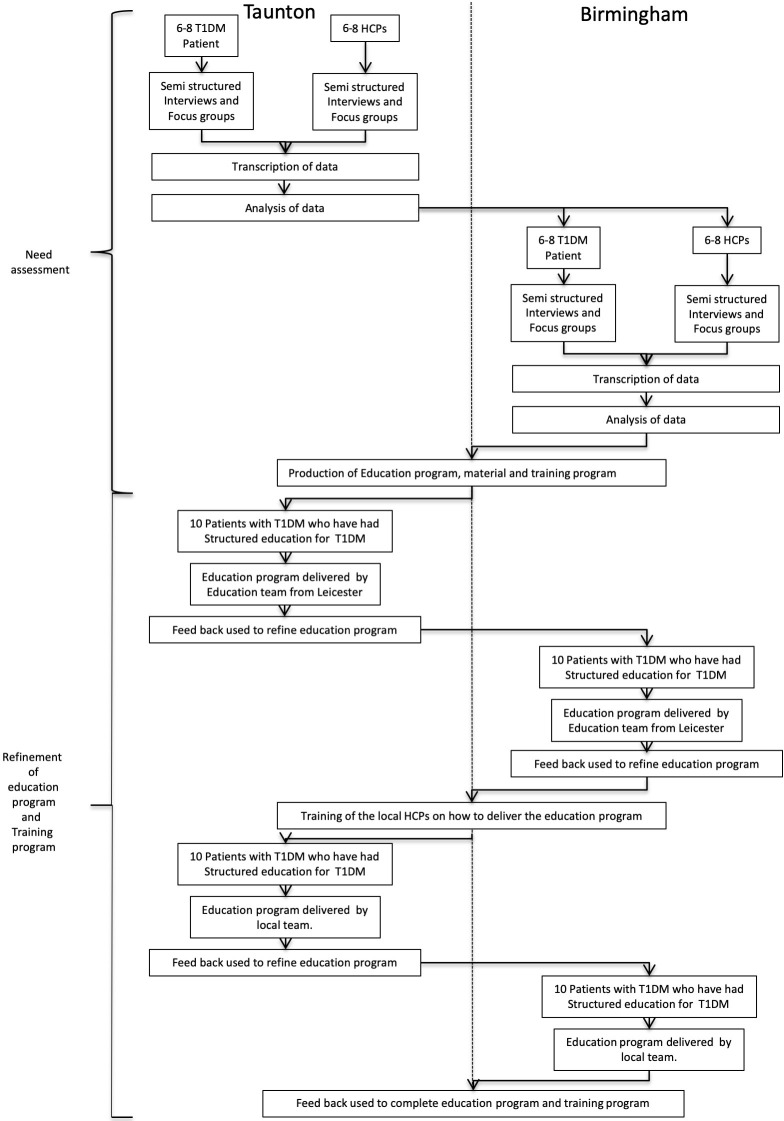
Flow of participants through phase 1. HCP, healthcare professional; T1DM, type 1 diabetes mellitus.

Phase 1 will involve the development of a structured group education program to provide adults with T1DM with the skills, knowledge and confidence to manage their diabetes before, during and after exercise (see figure 2 for overview). A multidisciplinary team (MDT) of experts in supporting exercise and PA for people with T1DM alongside researchers with experience of developing self-management education will develop an exercise program using the revised Medical Research Council (MRC) framework^13^ for developing a multicomponent intervention. The intervention will be underpinned with psychological theories previously demonstrated to promote behavior change and successfully used in other validated structured self-management programs for diabetes such as Diabetes Education and Self-Management for Ongoing and Newly Diagnosed^14^ and Dose Adjustment for Normal Eating (DAFNE).^15^ Key to these programs’ success is the attention given to educator training and the iterative development of the curriculum and supporting resources.

The process will begin with a literature review, followed by development of a written curriculum and supporting educational resources suitable for a broad range of participants with T1DM already engaging in regular PA. The content of this curriculum will be informed by exercise and diabetes experts, people with T1DM, and experts in developing structured education programs.

#### Literature review

Dietetic, diabetes management, physiological and other relevant data on T1DM and exercise/activity will be identified by a database search. Relevant searches will also be conducted on gray literature, including conference proceedings and protocols. Information from these sources will be collated, selected and summarized and used to identify potential topics for inclusion in the education program. Additionally, existing resources for both people with T1DM and HCPs will be considered, including web-based resources and our own research and clinical experience.

The education team, supported by clinical psychology guidance, will concurrently review the literature on psychological theories of learning to identify the most appropriate theories to underpin the developing intervention, consulting with colleagues working in the same field as part of the process.

#### Needs analysis

Semistructured interviews will be conducted with people with T1DM and with HCPs (dietitians, diabetes nurses, and diabetologists) likely to be delivering the education program, and other experts in the field. These interviews will help identify the needs of our stakeholders and shape the program structure, content, learning activities, supporting materials and resources, and educator training. Topic guides (online supplementary tables 1 and 2) will be used to guide the interview.

10.1136/bmjdrc-2019-000693.supp1 Supplementary data

Two focus groups, each of six to eight people, will be formed in Taunton and Somerset NHS Foundation Trust. One group will comprise people with T1DM and the second a multidisciplinary group of HCPs. Groups will be audio recorded with permission, and later transcribed for analysis using NVivo software. Thematic analysis of the focus groups will be informed by grounded theory.

Following analysis, a second set of two focus groups will be held in the University Hospitals Birmingham NHS Foundation Trust, again one group to comprise people with T1DM and the second a multidisciplinary group of HCPs. These groups will also be audio recorded and analyzed as above.

#### Development of the intervention

A curriculum development group will define the theories and philosophies to underpin the program, the potential outcomes and the target group. The specialist team will develop the detail of the intervention, and the accompanying training program for educators. This process will identify the performance indicators to be used to establish intervention fidelity of the prototype program during the pilot study. Concurrent with this development, the education team will work with the in-house designer to develop appropriate supporting resources and teaching aids.

The development team, having established the psychological and philosophical framework of the intervention, will take the learning and insights from the focus groups and combine this into a prototype intervention. This comprises stage 1 of the development. Stage 2 is formed of an iterative process described under development testing below.

#### Development testing

The development testing will undergo a minimum of four iterations, two at each of the centers (Taunton and Birmingham). For each iteration (and at each center), a group of 8–10 participants will be identified to take part in the program. At each center, an MDT of three HCPs will be identified as prospective educators of the new program. These HCPs will be experienced in delivering DAFNE or equivalent program to people with T1DM. This will be an iterative process as outlined in the online supplementary tables 1 and 2 in which the experiential learning of developers, participants and educators from each delivery event is fed back into the loop to produce the next version of the intervention.

### Methods for phase 2

Phase 2 will comprise a pilot RCT, designed in accordance with the Consolidated Standards of Reporting Trials guidelines. See figure 1 for a phase 2 overview.

#### Trial-specific tests and procedures

An overview of the visits for the study is shown in table 1. We aim to randomize 96 participants with T1DM to the study. Participants randomized to the usual care arm will be asked to attend six visits over a period of 36 weeks. Participants randomized to the intervention arm will be asked to attend additional visits for the education program (eight in total).

**Table 1:** Overview of visits for phase 2

Time (weeks)	Visit	Test/action	Usual care arm	Intervention arm
	1	Consent, screening (medical history, clinical examination (height, weight, waist circumference, body fat and blood pressure), HbA1c, FBC, U+Es, LFTs, C peptide, fit activity monitor and continuous glucose monitor (if applicable), complete questionnaires (Hypoglycemia Fear Survey, Clarke’s Hypoglycemia Awareness, BAPAD, EQ-5D, PAID and T1-DDS). Provision of blood glucose and insulin diaries.	✔	✔
	2	Collection of activity monitor and continuous glucose monitor, and blood glucose monitor and insulin diaries.	✔	✔
Randomization by a researcher between visits 2 and 3				
0–8	3	Education session 1	✔	✔
	4	Education session 2		✔
	5	Education session 3		✔
10–16	6	Joint nurse, doctor and dietitian appointment	✔	✔
26–33	7	6-month assessment, medical history, clinical examination (height, weight, waist circumference, body fat and blood pressure), HbA1c, FBC, U+Es, LFTs and diabetes-related autoantibodies, fit activity monitor and continuous glucose monitor (if applicable), complete questionnaires (Hypoglycemia Fear Survey, Clarke’s Hypoglycemia Awareness, BAPAD, EQ-5D, PAID and T1-DDS). Provision of blood glucose and insulin diaries.	✔	✔
28–34	8	Final visit. Collection of activity monitor and continuous glucose monitor (if applicable), and blood glucose monitor and insulin diaries.	✔	✔

BAPAD, Barriers to Physical Activity in Type 1 Diabetes; EQ-5D, EuroQol-5 Dimension; FBC, full blood count; LFT, liver function tests; PAID, Problem Areas in Diabetes scale; T1-DDS, Diabetes Distress Scale for Adults with Type 1 Diabetes; U+E, urea and electrolytes.

##### Visit 1 (consent and baseline test)

At this visit, written informed consent will be obtained. After consent, a clinical history and examination will take place. Routine blood tests and measure of C peptide will also be undertaken (table 2).

**Table 2:** Test/action/questionnaires conducted at baseline and 6 months

Clinical examination	Cardiovascular/respiratory/gastrointestinal/nervous system/feet
	Blood pressure and heart rate
	Height, weight, waist circumference
	Body fat content (bioimpedance)
Non-fasting blood	HbA1c
	FBC
	Liver/renal function
	GAD, IA-2 and zinc transporter autoantibodies (6-month assessment only)
	C peptide (baseline only)
Questionnaires	Clarke's Hypoglycemia Awareness Questionnaire
	The Hypoglycemia Fear Survey
	Gold score, Edinburgh hypo survey, and hypoawareness score
	Problem Areas in Diabetes (PAID) scale
	Diabetes Distress Scale for Adults with Type 1 Diabetes (T1-DDS)
	Barriers to Physical Activity in Type 1 Diabetes (BAPAD-1) scale
	EQ-5D
Purpose-designed diaries	Blood glucose monitoring diary
	Insulin monitoring diary
	Activity diary
Activity monitor	Small electronic device worn on wrist for 7 days. Measures physical activity by continually monitoring and recording movements of the body.
Hypoglycemia	4-week download of blood glucose monitor
Continuous glucose monitor	Wearing a monitor on stomach that continuously records glucose every 5 min for 7 days (optional)
Telephone call	Participants telephoned to inform them which treatment arm they had been allocated to
Clinical review	Review by nurse, dietitian and doctor to ensure no changes need to be made to insulin dosages or medication
Participant postcard	Postcard given at last visit for participant to provide anonymous feedback on the study

EQ-5D, EuroQol-5 Dimension; FBC, full blood count; GAD, glutamic acid decarboxylase; IA-2, islet tyrosine phosphate 2.

Data will be collected to explore how best to measure this outcome. These will include questionnaires, a structured, purpose-designed, glucose and insulin monitoring diary, blood glucose monitor and optional 7-day continuous glucose monitoring system. The data from the continuous glucose monitoring system will be analyzed using international consensus guidelines to explore how outcomes relating to glucose variability are influenced by the education intervention.^16^

All participants will be given an activity monitor to wear for 7 days as well as a diary to record their activities for a week.

Questionnaires outlined in table 2 will be completed.

##### Visit 2

The activity monitor, continuous glucose monitor (if applicable) and blood glucose and insulin diaries will be collected. Following the visit, and once the results of all the investigations from visit 1 and visit 2 have been collated, the participant will be randomized by the research nurse or delegate.

##### Randomization and intervention

Participants will be randomized through a web-based randomization system (sealed envelope), which will be managed by Leicester Clinical Trials Unit (LCTU). Participants will be assigned in a 1:1 ratio, to usual care (an update of their local T1DM education course, eg, DAFNE, Beta Cell Education Resources for Training in Insulin and Eating (BERTIE), Living with Diabetes) or the intervention (Exercise for Type 1 Diabetes (EXTOD) Education program). Although the protocol stated that randomization will be minimized on center, activity levels, gender and number of hypoglycemia events per month, the randomization will in fact be minimized on center, average minutes of moderate activity per week, gender and average number of hypoglycemia events per week. Due to the nature of the intervention, blinding of the participants or the study team to the randomization arm is not possible.

##### Visits 3, 4, and 5

Participants allocated to the intervention arm will attend the new EXTOD Education course. This will consist of three half-day sessions spread over an 8-week period. At the first session the participants will introduce themselves, outline what exercise they are currently doing and the problems that they are experiencing in terms of glucose control. They will also be encouraged to identify questions that they want to be answered during the education program. The physiology of glucose control at rest and during exercise will subsequently be discussed. Participants will be taught how to identify the type and intensity of exercise they are doing and also learn how to stay safe while exercising. The final part of this session will teach them three strategies to control their glucose during exercise. These strategies are (1) changing mealtime insulin, (2) taking carbohydrate while exercising, and (3) adapting the intensity and type of exercise. Participants will be encouraged to test these strategies before the next session.

The second session will commence with the participants describing their progress with the strategies taught at the first session. Managing diabetes following exercise will subsequently be discussed. This will include post-exercise physiology, daily protein and carbohydrate requirements for exercise and the types of food and fluid that should be taken before, during and after exercise. The final part of this second session will teach participants the three strategies to manage glucose after exercise. These will be changing fast-acting and background insulin, carbohydrate and protein fuelling and adapting their warm down. Participants will be encouraged to test these new strategies before the final session.

At the third and final session participants will once again be encouraged to provide feedback on events since their previous session. Complex case studies will then be discussed, for example, how to manage glucose during prolonged endurance events. At the end of this session we will signpost them to additional material and the study website. On this website the participants will be able to review the information given on the program, get more detailed information about how to manage specific sports and explore more case studies.

A specialist diabetes nurse, a specialist diabetes dietitian and a doctor who specializes in diabetes will deliver the structured education program. As per national guidelines for structured education programs, educators will use an evidence-based curriculum and core set of resources for delivery. This will ensure that there is consistency in content and delivery style across sites. Educators will use a mixture of methods to deliver the program such as visual displays, group work and problem solving. In addition the participants will be given a personal handbook covering the content of each of the sessions, for reference at a later date. During the study, to ensure treatment fidelity, the study team will video record some of the education sessions to be delivered.

A postcard will be provided to participants to obtain additional feedback on the education sessions.

##### Visit 6

Participants in both study arms will have an appointment with the MDT that delivered their education session. In the session the team will look at overall diabetes care as well as focusing on glucose control around exercise.

##### Visit 7

The participant will see the research nurse/researcher for their 6-month assessment. All the assessments done at baseline will be repeated, except C peptide measurement, with an additional blood sample to measure diabetes-related autoantibodies (table 2).

##### Visit 8

The participant will see the research nurse/researcher. The activity monitor, continuous glucose monitor and blood glucose and insulin diaries will be returned by the participant.

The duration of all visits will be recorded to inform future health economic evaluations for the definitive RCT.

##### Statistical analysis

The proportion of eligible participants who consent to participate in phase 2 of the pilot trial will be presented by center and overall, along with the proportions in each treatment group completing each follow-up assessment and the reasons for withdrawal. Descriptive characteristics and outcome data will be summarized overall and by treatment group, as mean (SD) for symmetrically (eg, normal) distributed continuous variables, median (IQR) for skewed continuous variables, and number (percentage) for categorical variables. As this is a pilot study there will be no formal comparisons between interventions.

### Study population and recruitment

Study inclusion criteria will include participants with: a clinical diagnosis of T1DM, aged 18–70 years, hypoglycemia awareness, knowledge of carbohydrate counting, using a basal bolus regime, completion of nationally accredited structured education program and doing at least 30 min of exercise twice a week or signed up to a sporting event to take place in the next 3–6 months. Participants will be excluded if they are pregnant, using an insulin pump, have hypoglycemia unawareness, are unable to exercise, understand English or give informed consent.

Participants will be recruited from secondary care sites, using lists of existing patients, those interested in research and clinical databases. Additionally, participants will be recruited from primary care using general practitioner (GP) databases, retinal screening clinics, specialist nurse clinics, through direct contact with HCPs, medical professionals and pharmacies. Additionally, direct marketing/open self-referral will be used to ensure that as wider selection of potential participants as possible can be reached. Posters will be displayed in places such as pharmacies, community centers, libraries, and large employers (eg, university and hospitals) in order to engage with the potential population.

### Study setting

The study will be conducted at two hospitals in the UK: Taunton and Somerset NHS Foundation Trust and University Hospitals Birmingham NHS Foundation Trust. The first is a medium-sized hospital in the Southwest that provides care for people living in a mixture of rural and urban environments and the latter is a large teaching hospital in the West Midlands that provides care for people living predominantly in an urbanized metropolitan environment.

### Ethics and dissemination

The study protocol was approved by West Midlands-Coventry and Warwickshire Research Ethics Committee (REC) (reference 16/WM/0034) and the study was registered on ISRCTN on 27 July 2016. Taunton and Somerset NHS Foundation Trust has agreed to act as sponsor for the study. The study will conform to the International Conference on Harmonisation of Good Clinical Practice guidelines as well as the Declaration of Helsinki. Written informed consent will be obtained from each patient before any study-specific procedures are performed. A Trial Steering Committee, comprising independent experts and lay members, will be established to provide overall supervision of the progress and conduct of the trial, as well as advice on scientific credibility. Those allocated to the usual care arm will be offered the chance to attend the education program when the study ends.

The findings from the study will be disseminated by usual academic channels, that is, presentations at international meetings, as well as by peer-reviewed publications and through patient presentations and newsletters to patients, where available. Publication or presentation of the results will be in line with the funder agreement and intellectual property guidelines and with the permission of the CI.

### Trial management

The study will be managed by the recruiting sites at Taunton and Birmingham, and supported by LCTU, a UK Clinical Research Collaboration accredited Clinical Trials Unit (CTU). The LCTU team will manage a web-based randomization system, develop and maintain the trial database, check data quality as the trial progresses, carry out statistical analyses in collaboration with the clinical investigators, and liaise with and support staff at each of the recruiting sites.

### Adverse events

All adverse events (AE) occurring during the study observed by the investigator or reported by the participant, whether or not attributed to the study, will be recorded and tracked through to resolution. The relationship of AEs to the study will be assessed by a medically qualified investigator. All serious adverse events (SAE) will be reported within 24 hours in line with the sponsor (or delegate) standard operating procedures. All suspected unexpected serious adverse reactions (SUSAR) will be reported to the REC within the stated time period. In addition to the expedited reporting above, the chief investigator (CI) shall submit once a year throughout the study, or on request, an annual report to the REC which lists all SAEs/SUSARs that have occurred during the preceding 12 months.

Advice on carbohydrate and insulin dose adjustment around exercise will be prepared and distributed to both HCPs and patients. The study team will keep in contact with participants as they start and increase their exercise intensities so that the risks of hypoglycemia and injury can be minimized. Any such events will be documented and reviewed at the study meetings. Addressing this risk is an important aspect of this trial. The research team will therefore either provide standard advice (which is currently being provided to patients when they exercise), or advice developed with the education program.

### Trial status

Recruitment to phase 1 (development of the education program) commenced in June 2016, and to phase 2 (pilot RCT) in August 2017. Follow-up of the last patient for phase 2 was completed in February 2019.

The findings from the needs analysis undertaken to inform the education package have been published in a peer-reviewed journal in February 2019.^17^ This outlines patient and HCP perspectives on the delivery of exercise education for patients with T1DM. The development and outline of the structured education program was under review at the time this article was prepared.

## Discussion

Regular exercise in people with T1DM can result in considerable improvements in health and reduction in cardiovascular events and death. However, a large proportion of people with T1DM are not active. Fear of hypoglycemia and lack of knowledge on how to manage their diabetes are major barriers to exercise in people with T1DM, but few patients receive specific advice about how to adjust insulin and carbohydrate for activity. These issues are not addressed in generic guidelines around exercise nor around management of T1DM. There is a recognized need for such guidelines because HCPs currently lack the knowledge to advise patients on how to manage their diabetes when active and would like formal training in exercise prescription for people with T1DM.

This article outlines the protocol for a pilot RCT to develop a program of education that will support adults with T1DM to undertake safe and effective exercise. This is accompanied by training for HCPs to provide this education. The work is divided into two stages. The first stage develops an education program using the MRC framework. The second stage is a pilot RCT that aims to collect the key variables to design a definitive trial to test the efficacy and cost-effectiveness of the education package.

Successful completion of this program of work will generate knowledge translation resources that will be useful for HCPs and patients, address barriers to exercise in adults with T1DM and should facilitate an increase in exercise for this group of people.
