# Supplementary material for: Rationale and methods for the Exercise for Type 1 Diabetes Education program: a pilot randomized controlled trial of an education program to support adults with type 1 diabetes mellitus (T1DM) to undertake exercise
Source: BMJ Open Diabetes Res Care. 2019 Dec 30;7(1):e000693. doi: 10.1136/bmjdrc-2019-000693 (PMC6954769; doi:10.1136/bmjdrc-2019-000693)
Supplement: Supplementary data [file bmjdrc-2019-000693supp001.pdf]

Supplementary table 1: Participant interview topic guide for feedback

Thank you for staying to answer my questions and provide feedback on your experience of the EXTOD Education pilot. I am going to start with some general questions and then there will be some that are more specific.

Overall session feedback

- What did you think of the sessions?
- Did the sessions meet your expectations? (Yes/no – in what way? Please give examples)
- How did you feel about the number of sessions and the spread of these over 6 weeks?
- How did you feel about the length and timing of the sessions?
- What key messages did you take away from the programme?
- What are your thoughts about the group size now that you have experienced the programme?
- Was it helpful having the option to bring someone with you?

Session specific

- How did you find session 1?
- What did you think of the resources that supported the content? (Norm and Tod; cycle ride; aerobic/anaerobic exercise; hypo game; traffic light game; High/Low GI; Strategies Pie Chart)
- How did you find the handbook? What changes would you make to it?
- During the first session, a doctor came to talk to you, how did you find that?
- How important is it to have a doctor as part of the sessions? What content would you like them to cover?
- How did you find the content in sessions 2 and 3?
- What, if anything, did you feel was missing from the 3 sessions?

Improvements/developments

- How would you feel about having a practical element to the programme, for example, taking part in a brisk 30-minute walk?
- If we were to develop an online-resource to accompany the face to face sessions, what should we consider? What should we include?

- *What advice do you have for us to promote the study and the education programme?*

Your management/impact

- *Has anything changed in how you manage exercise and your diabetes since attending ExTOD? If so, what are you doing differently?*
- *What role has ExTOD had in making these changes? Would you have made them without coming?*

Thank you for your time and input. Your comments are invaluable to the development process.

Supplementary table 2: Healthcare professional interview topic guide for feedback

Overall session feedback

- What did you think of the sessions?
- How did you feel about the number of sessions and the spread of these over 6 weeks?
- How did you feel about the length and timing of the sessions?
- What are your thoughts about the group size?
- What are your thoughts on the option for people to bring someone with them?

Session specific

- How did you find session 1?
- What did you think of the resources that supported the content? (Norm and Tod; cycle ride; aerobic/anaerobic exercise; hypo game; traffic light game; High/Low GI; Strategies Pie Chart)
- How did you find the handbook? What changes would you make to it?
- How did you find the content in sessions 2 and 3?
- What, if anything, did you feel was missing from the 3 sessions?

Improvements/developments

- How would you feel about having a practical element to the programme, for example, taking part in a brisk 30-minute walk?
- If we were to develop an online-resource to accompany the face to face sessions, what should we consider? What should we include?
- What advice do you have for us to promote the study and the education programme?

Thank you for your time and input. Your comments are invaluable to the development process.
